# The emergence of super-resolution beyond the probe size in scanning 3DXRD microscopy

**DOI:** 10.1107/S1600577523008597

**Published:** 2023-10-17

**Authors:** Jaemyung Kim, Yujiro Hayashi, Makina Yabashi

**Affiliations:** a RIKEN SPring-8 Center, 1-1-1 Kouto, Sayo-cho, Sayo-gun, Hyogo 679-5148, Japan; b Japan Synchrotron Radiation Research Institute, Sayo-cho, Sayo-gun, Hyogo 679-5148, Japan; University of Tokyo, Japan

**Keywords:** scanning 3DXRD, super resolution, beam trajectory

## Abstract

The spatial resolution in scanning-based two-dimensional microscopy is normally limited by the size of the probe, thereby a smaller probe is a prerequisite for enhancing the spatial resolution. For three-dimensional microscopy that combines translation and rotation motions of a specimen, however, complex trajectories of the probe highly overlap in the specimen, which could change the postulate above. Here, the spatial resolution achieved in scanning three-dimensional X-ray diffraction (s3DXRD) microscopy is investigated. In this method, the most appropriate orientation of the pixel in the specimen coordinate is selected by comparing the completeness of diffraction peaks with theory. Therefore, the superposed area of the beam trajectory has a strong effect on the spatial resolution, in terms of the completeness of diffraction peaks. It was found that the highly superposed area by the incident X-rays, which has the highest completeness factor in the pixel of the specimen, is much smaller than the X-ray probe size, and that sub-pixel analysis by dividing a pixel into small pieces leads to drastic improvement of the spatial resolution in s3DXRD.

## Abstract 2

It has been believed that the resolution in scanning-based microscopy is defined by the probe size, but this postulate is not valid in scanning three-dimensional X-ray diffraction (3DXRD) microscopy that combines the translation and rotation motions of a specimen. Numerical simulation shows that the spatial resolution in scanning 3DXRD microscopy surpasses the probe size due to the superposition of the X-ray beam trajectories.

## 1. Introduction

In a microscopic analysis, spatial resolution refers to the ability of the imaging system to distinguish closely separated objects. In optical microscopy, resolving power is typically measured by the point spread function (PSF) (Cole *et al.*, 2011 ▸) or the modulation transfer function (MTF) (Giger & Doi, 1984 ▸). The PSF describes the shape and spread of a point source of light as it is imaged by the optical system, while the MTF characterizes how well the optical system reproduces spatial frequencies in the image. In scanning-based two-dimensional (2D) microscopy, the spatial resolution is predominantly determined by the size of the probe (Nguyen *et al.*, 2001 ▸; Bian *et al.*, 2021 ▸). Therefore, a smaller probe size is a promising solution for achieving both a high spatial resolution and a high signal-to-noise ratio. As the probe size decreases, smaller probes in the field of view give more detailed information, resulting in higher-resolution images.

As a scanning-based microscopy technique, scanning three-dimensional X-ray diffraction (s3DXRD) microscopy is a powerful tool for the non-destructive investigation of the micro-structure of grains such as orientation and shape (Hayashi *et al.*, 2015 ▸, 2019 ▸). The most probable orientation in the pixel of the specimen coordinate is selected from the candidates by comparing the number of detected peaks *N* with the number of theoretical peaks *M*, which is known as multigrain indexing (Sørensen *et al.*, 2012 ▸; Wejdemann & Poulsen, 2016 ▸). The grain orientation map is obtained by finding the largest completeness factor *N*/*M*, denoted as *N*′, over the specimen coordinates (Hayashi *et al.*, 2015 ▸). Since the grain with the highest *N*′ is always illuminated by X-rays under any rotation angle, the best grain candidate can be selected in this manner.

In s3DXRD microscopy, the data are analyzed with the assumption that the spatial resolution is approximately twice the size of the probe (Hayashi *et al.*, 2015 ▸; Henningsson *et al.*, 2020 ▸). However, the incident X-ray beam has complex trajectories in a rotated specimen, and the superposed confined area of the beam trajectory is generally much smaller than the original beam size. If we apply the *N*′ analysis in the multigrain indexing to the small confined area, we could effectively improve the spatial resolution in s3DXRD far beyond the original beam size. In this study, we performed a simulation and found that the superposition of the X-ray beam trajectories effectively reduced the confined area to about one-fifth of the probe beam size, and proved that the effective spatial resolution in s3DXRD can be enhanced by a factor of five.

## 2. Rotation of specimen in s3DXRD

**Figure 1:**
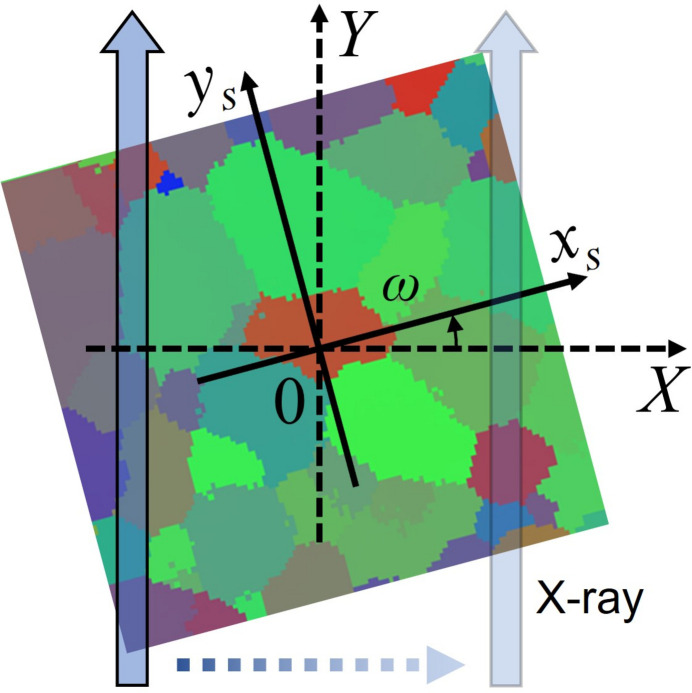
Scanning procedure in s3DXRD microscopy. The diffraction peaks are measured by translating and rotating the specimen. The rotation center is unchanged during the measurement.

A grain orientation map of polycrystalline materials can be obtained by analyzing the diffraction peak position over the reciprocal space. By comparing and indexing the peak positions belonging to an arbitrary specimen coordinates with the theoretical values, the most probable grain orientation candidate at each coordinate can be selected. Therefore, it is essential to prepare a diffraction data set penetrating a specimen coordinate (*x*_s_, *y*_s_) under different angles. As illustrated in Fig. 1 ▸, in s3DXRD the diffraction data set is obtained by combining the translation and rotation of the specimen. In the real measurement, the incident X-ray beam position is unchanged, while the sample moves perpendicular to the X-ray beam directions. At each translational step, the sample rotates by ω from 0 to π. These operations can be described with specimen coordinates (*x*_s_, *y*_s_) as follows,

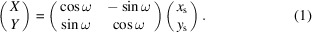

Since *X* = 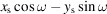 , *X* is not always an integer. By rounding *X* to the nearest number, we get the (*X*, ω) list for a pixel (*x*_s_, *y*_s_). In the case of *Y*, it is determined by the ω list at (*x*_s_, *y*_s_). We can ignore this value since *Y* does not affect the simulation. The matrix expression given in equation (1) is consistent with the data selection procedure proposed by Hayashi *et al.* (2015 ▸).

## 3. Completeness factor in s3DXRD

The main purpose of s3DXRD is to obtain the orientation map of polycrystalline materials. From the diffraction peak positions, we get **G** vectors at (*x*_s_, *y*_s_) from the (*X*, ω) list. Each pixel position (*x*_s_, *y*_s_) is considered as an independent crystal. Since at least one crystal is always rotating around (*x*_s_, *y*_s_), we can find the most probable orientation among the candidates by comparing the completeness factor *N*′, where *N*′ is the ratio between the number of observed peaks and the theoretical peaks, because the rotation point (*x*_s_, *y*_s_) has the highest number of diffraction peaks or highest completeness factor *N*′ close to 1. In the s3DXRD measurement, the sample rotates at a constant speed, and the diffraction spots are integrated over the angular interval. The diffraction peaks can be detected at certain angles if the lattice orientation satisfies the Laue condition. Due to the continuous rotation motion, *M* becomes a constant value. Consequently, *M* remains fixed when the orientation is determined.

## 4. Numerical simulation

To understand the effect of the superposition of the X-ray beam on *N*′, we performed a numerical simulation. For the simulation, we set the *X* step range to ±10 with an increment of 1, and the angular step to 10°. To avoid the complexity of the figure by X-ray beam overlapping, the angular step was intentionally adjusted to 10°. We set a zero matrix of 440 × 440, then illuminated X-rays belonging to a pixel (*x*_s_, *y*_s_) by marking the illuminated area to 1. The X-ray beam width and its translational step were set to 40 pixels and 40 pixels, the same as the X-ray beam size, respectively. We assumed that the beam shape is a box-shaped function. After that, we rotated the image matrix according to equation (1). We added +1 to the X-ray beam illuminated area and rotated the image matrix. By summing the matrices at every angle penetrating the pixel (*x*_s_, *y*_s_), we obtained the completely superposed position. After converting the units of (*x*_s_, *y*_s_)-coordinates into mm units (40 pixels = 1 mm), we obtained a superposed map of the incident X-rays of 1 mm.

**Figure 2:**
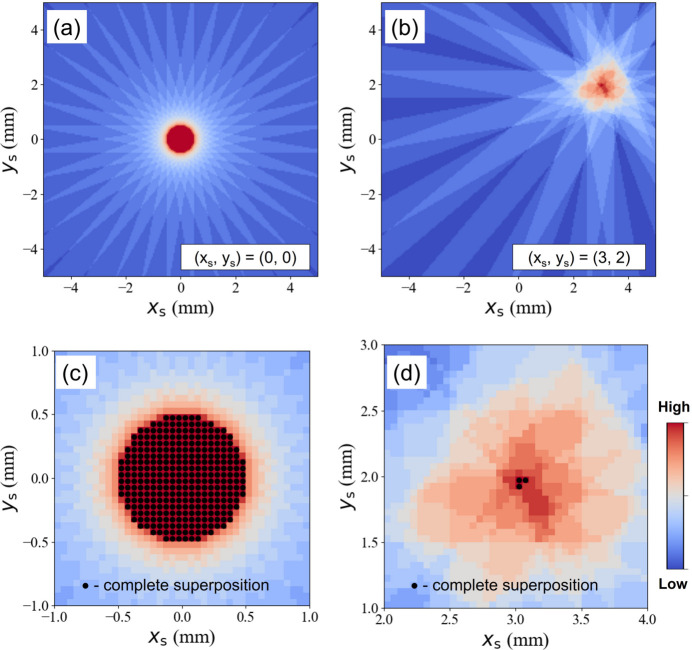
Beam trajectories at (*x*_s_, *y*_s_) = (0, 0) and (3, 2). (*a*) The incident X-rays are perfectly illuminating the rotation center where symmetric beam trajectories are seen. (*b*) X-ray beam trajectories for (*x*_s_, *y*_s_) = (3, 2) show that the X-rays are not perfectly aligned to (*x*_s_, *y*_s_) = (3, 2) but partly shining on the target point, which confines the X-ray illumination to a small area. Zoom-ins on the completely superposed region for (*c*) (*x*_s_, *y*_s_) = (0, 0) and (*d*) (*x*_s_, *y*_s_) = (3, 2) are illustrated.

Figure 2 ▸ shows the numerical simulation results on (*x*_s_, *y*_s_) = (0, 0) and (3, 2). From Fig. 2 ▸(*a*), we find that the X-rays are perfectly penetrating the pixel (0, 0) with the ideal rotation center. Due to the finite X-ray beam size, the diameter of the superposed circle is the same as the beam width. In this case, the completeness factor *N*′ of the multigrain indexing will be the same over this circular region. Therefore, the spatial resolution at this point is the same, and is defined by the probe size, since the crystallographic orientations in this area will be all the same. Figure 2 ▸(*c*) shows a zoom-in of the center part of Fig. 2 ▸(*a*). The black closed circles show the fully superposed area, whose overall envelope is a circle composed of 316 pixels. On the other hand, if we move to another pixel in the specimen coordinate, *e.g.* (*x*_s_, *y*_s_) = (3, 2), the fully superposed area is reduced to 3 pixels as shown in Figs. 2 ▸(*b*) and 2(*d*). At this coordinate, the X-ray beam center is not perfectly heading to (*x*_s_, *y*_s_) = (3, 2) but illuminates the point with some part of the beam. Therefore, the area having the highest *N*′ in the multigrain indexing is more confined at (*x*_s_, *y*_s_) = (3, 2), which can provide high spatial resolution. This idea can be extended for the high-spatial-resolution orientation map in s3DXRD.

**Figure 3:**
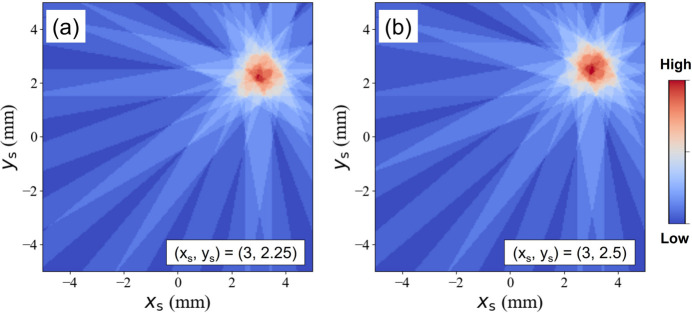
Beam trajectories illuminating (*a*) (*x*_s_, *y*_s_) = (3, 2.25) and (*b*) (*x*_s_, *y*_s_) = (3, 2.5), close to (3, 2), show a different feature.

In s3DXRD, fractional coordinates have not been introduced yet, since there has been no consideration of *N*′ and the superposition of X-rays. However, according to the present simulation, the introduction of fractional coordinates can increase the spatial resolution, in terms of *N*′ during the multigrain indexing. Fractional coordinates can be obtained by sub­dividing a pixel into small pieces, called a *sub-pixel*. With this method, we can obtain a data set for (*x*_s_, *y*_s_) = (3, 2.25), which is near (3, 2) but has a slightly different diffraction data set as illustrated in Fig. 3 ▸(*a*). Let us assume that the image sets required for (3, 2) and (3, 2.25) are [Img(*x*_0_, *ω*_0_), Img(*x*_1_, *ω*_1_), Img(*x*_2_, *ω*_2_), Img(*x*_3_, *ω*_3_), Img(*x*_4_, *ω*_4_)] and [Img(*x*_0_, *ω*_0_), Img(*x*_1_, *ω*_1_), Img(*x*_2_, *ω*_2_), Img(*x*_4_, *ω*_3_), Img(*x*_5_, *ω*_4_)], respectively. The two neighboring positions share three images [Img(*x*_0_, *ω*_0_), Img(*x*_1_, *ω*_1_), Img(*x*_2_, *ω*_2_)], but the other two images are different. This indicates that the **G** vectors from the three images are the same but the other two images give different values. In the multigrain indexing process, the error in the **G** vectors is minimized by adjusting the orientation matrix. Therefore, the two different images have an effect on the optimization of the orientation matrix. This gives a slightly different optimized orientation if the two points (3, 2) and (3, 2.25) belong to the same grain. In the same manner, this method can be applied to (3, 2.5) as shown in Fig. 3 ▸(*b*). The slightly different X-ray beam trajectories confine the X-ray illumination to (3, 2.5), which is different from that of other coordinates. Therefore, the introduction of the sub-pixel will attribute to the resolution enhancement in the orientation map, except for the rotation center.

**Figure 4:**
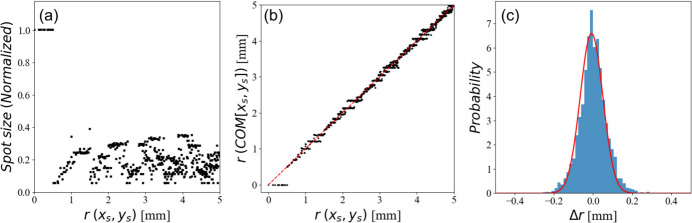
The radial average of the relative spot size of the fully superposed area shows an increase in spatial resolution (*a*). The average of the spot is calculated to be about 0.2, which is an indication of the five times increase in the spatial resolution. The relation between the radius from the specimen coordinates and the center-of-mass position of the fully superposed area is illustrated in (*b*). The red-dotted line shows a linear relationship between the two. A histogram of the differences between the two variables with Gaussian fit is shown in (*c*). The FWHM of the fit is about 0.136, which indicates a positional error of ±6.8% concerning the incident X-ray beam size.

We conducted a simulation on sub-pixels by dividing each pixel into 4 × 4 pieces in 2D space, for whose each fractional coordinates are 0, 0.25, 0.5 and 0.75 in the (*x*_s_, *y*_s_) plane. The superposed area was calculated over the specimen coordinates for the investigation of resolution and normalized with the beam size. As described in Fig. 4 ▸(*a*), the average spot size is reduced to about 20% except for the center of rotation. This implies a five times increase in the spatial resolution, in terms of *N*′ in multigrain indexing. The center of mass (COM) of the completely superposed area is compared with the ideal pixel position *r*(*x*_s_, *y*_s_) for the evaluation of the accuracy as shown in Fig. 4 ▸(*b*), where *r*(*x*_s_, *y*_s_) and *r*(COM[*x*_s_, *y*_s_]) indicate the radius of the selected pixel and the COM of the fully superposed area belonging to the pixel, respectively. The red-dotted line shows a linear relationship between the two. Except for *r* < 0.5, *r*(*x*_s_, *y*_s_) and *r*(COM[*x*_s_, *y*_s_]) show a linear relation with small deviation, which indicates a low positional error. The X-ray diffraction image data set for the multigrain indexing is all the same for any *r* < 0.5, although we included fractional coordinates. A histogram of the difference between *r*(*x*_s_, *y*_s_) and *r*(COM[*x*_s_, *y*_s_]) is depicted with the fit in Fig. 4 ▸(*c*). The evaluated FWHM (full width at half-maximum) is about 0.136. This value indicates that the positional error is ±6.8% of the incident X-ray beam size. The center of mass of the perfectly overlapped position deviates from the target position (*x*_s_, *y*_s_). As a result, the highest *N*/*M* is not always from (*x*_s_, *y*_s_) but away from the position by ±6.8% of the beam size. Consequently, even though the perfectly overlapped area is significantly smaller than the size of the X-ray beam, the resolution enhancement will be limited due to the presence of the positional error.

**Figure 5:**
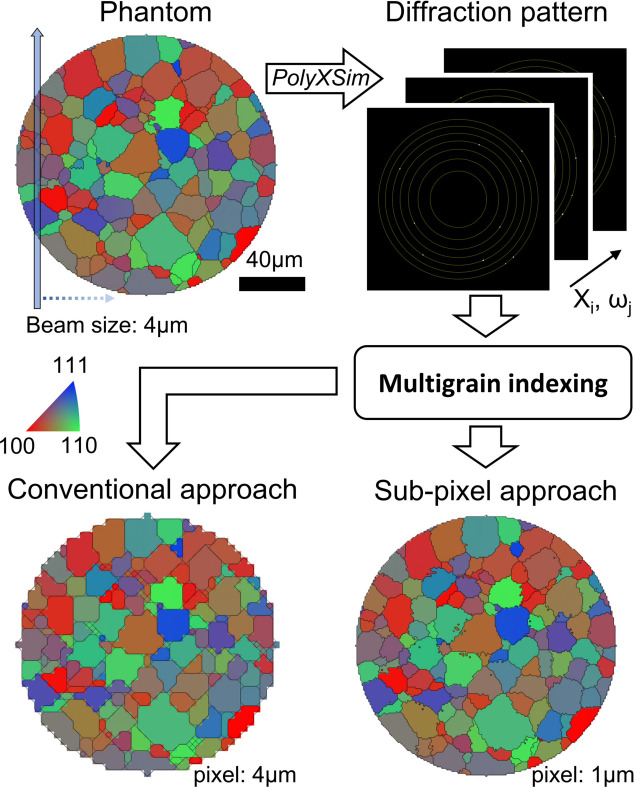
Reconstruction simulations on the phantom map using the conventional approach and the sub-pixel approach. The virtual X-ray beam size was set to 4 µm, the same as the translation step of the phantom. Calculated X-ray diffraction images by *PolyXSim* were used as input images for the multigrain indexing. The reconstruction simulation result with the proposed sub-pixel approach shows more detail than the conventional approach.

In order to confirm the validity of the proposed sub-pixel approach, we have implemented a reconstruction simulation on the phantom map shown in Fig. 5 ▸. The diameter of the phantom was set to 160 µm. The virtual X-ray beam size was set to 4 µm and the X-ray beam translation step was 4 µm. By rotating the phantom in 0.5° steps, we calculated the diffraction patterns on the phantom, which meet the virtual X-rays. We assume that each pixel of the phantom is an independent crystal. For the simulation, we calculated diffraction patterns using *PolyXSim* in the *FABLE-3DXRD* software package (https://sourceforge.net/p/fable/wiki/PolyXSim/). The obtained diffraction images of the phantom map were used as input images for the 2D orientation map. By implementing multigrain indexing on the generated images we obtained 2D orientation maps. From the conventional approach the pixel size in the reconstructed image is the same as the specimen translation step, which results in a 4 µm pixel size as shown in Fig. 5 ▸. Although this approach gives rough orientation information, the detailed shapes of the grains deviate from the original phantom. On the contrary, the reconstruction result with the proposed approach provides much improved agreement with the phantom, which indicates the effectiveness of our new method.

**Figure 6:**
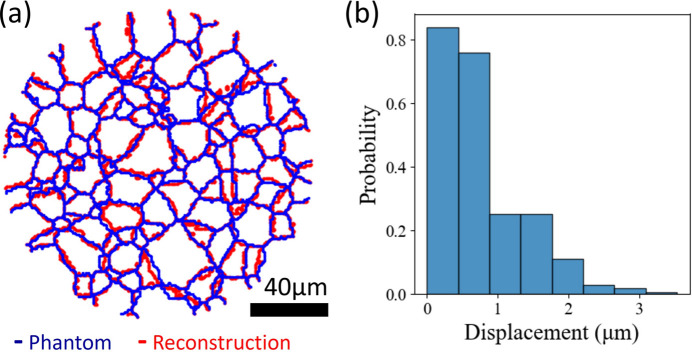
Overlay of grain boundary maps from the phantom and reconstruction simulation with 1 µm sub-pixel (*a*), and a histogram of the grain boundary displacement (*b*). The positional shift of the grain boundary is smaller than the X-ray beam size. The most probable displacement is smaller than 1 µm, and the weight averaged value is about 0.7 µm.

To quantitatively estimate the resolution enhancement of the proposed method, we extracted the grain boundary from the phantom and reconstruction simulation as shown in Fig. 6 ▸(*a*). The grain boundaries were extracted by thresholding the misorientation threshold to 3°. We can see that the grain boundaries are highly overlapped, although there are some mismatches due to artifacts. The displacement of the grain boundary position was evaluated from the minimum distance of the selected phantom’s grain boundary with the reconstruction simulation. Figure 6 ▸(*b*) shows a histogram of the displacement, and shows that the most probable displacement is below 1 µm and the maximum displacement is smaller than the X-ray beam size. The weighted average of the displacement is about 0.7 µm, smaller than the incident X-ray beam size of 4 µm. Therefore, if we evaluate the resolution enhancement from the average displacement, the value is about 5.7 times that defined by the X-ray beam. This indicates that the sub-pixel approach can enhance the spatial resolution, although it does not perfectly recover the grain shape.

## 5. Discussion

The superposition of the X-ray beam trajectories is influenced by the angular step in rotation operation. As the rotation step decreases, the superposed area becomes smaller, which increases the spatial resolution. Under a large rotation step, the superposed spot size is still smaller than the beam size. However, the number of diffraction spots observed in the s3DXRD will be decreased, which induces a decrease in *N*′, because the possibility of detection of the diffracted X-rays is low under a large rotation step. Even if the diffraction spots are measured by continuously rotating the sample, a large angular step increases the uncertainty of the rotation angle which results in an orientation map with large errors. Therefore, selecting the appropriate angular step is an important factor in s3DXRD microscopy.

Another parameter that affects the X-ray beam superposition is the incident X-ray beam size. In the present numerical simulation, the X-ray beam size was set to be the same as the increment of the specimen translation, which was found to be effective for achieving high resolution. If the X-ray beam size is larger than the increment of the specimen translation, it will cause an increase in the fully superposed area. As a result, the spatial resolution will decrease because the highest *N*′ is not confined to a small area. On the other hand, if the X-ray beam is smaller than the increment of the specimen translation, the diffracted X-ray beam does not always contain information of the desired position (*x*_s_, *y*_s_). In this case, the narrow X-rays will illuminate around the pixel (*x*_s_, *y*_s_).

Therefore, it is important to adjust the size of the X-ray beam to match the increment of the specimen translation as closely as possible. In real situations the X-ray beam is not box-shaped and has a tail. Even in this case the fully overlapped area of an arbitrary point is still smaller than that at (0, 0). The fully overlapped area will be larger than our expectation that the resolution enhancement will not be large but still better than that at (0, 0).

The super-resolution in our approach is distinguished from the conventional interpolation methods (Hajnal *et al.*, 1995 ▸) or machine-learning-based methods (Schulter *et al.*, 2015 ▸; Dong *et al.*, 2016 ▸). In conventional methods the new values are inferred from the existing output data. Therefore, the new data points are correlated with the old ones. However, in the s3DXRD microscopy case, the superposition of the X-ray beam automatically provides super-resolution, because the data set for the two neighboring specimen coordinates are slightly different. Therefore, the orientation of the two points is slightly different, which enhances the spatial resolution except for around the rotation center. Distinguished from the conventional interpolation methods or machine-learning-based methods, our approach requires different input data for resolution enhancement.

The number of specimen translation steps that Hayashi *et al.* (2015 ▸) and Hektor *et al.* (2019 ▸) selected in s3DXRD was 21 and 129, respectively. Under those conditions they were able to successfully reconstruct orientation maps. In those cases, the low-resolution areas around the rotation center are only 0.25% and 0.006%, respectively, which are negligibly small. This indicates that, if the number of specimen translation steps is enough, the low resolution around the rotation center may not be problematic.

However, this assumption is not always valid. For example, we could observe an ‘artifact’ diffraction signal even from a hole or void (*i.e.* the area without a crystal) in the target position. In addition, the grain shape needs to be a convex polygon, not a concave polygon, which is the limitation of the s3DXRD or reconstruction technique.

## 6. Conclusion

We present a breakthrough in the resolution limit of s3DXRD microscopy, which enables super-resolution beyond the probe size. By combining the principle of multigrain indexing with the superposition of X-ray beam trajectories, we show that the superposed area of the beam trajectory has a strong effect on spatial resolution in terms of the completeness of diffraction peaks. Our simulation results show that the completely superposed X-ray spot size is smaller than the incident X-ray beam size, which increases the spatial resolution in s3DXRD. The reconstruction simulation result on the phantom showed good agreement with our proposed approach. This breakthrough in s3DXRD microscopy will greatly extend our understanding of polycrystalline materials.
